# Acute Stroke Care in a Thrombolysis-Capable Emergency Department in Iraq: Patient Characteristics, Risk Factors, and Reperfusion Metrics From a One-Year Registry

**DOI:** 10.7759/cureus.111782

**Published:** 2026-06-29

**Authors:** Mustafa Al-Shahni, Israa Alrubaee, Ali S Yahya, Saad Kazim, Aktham El-Rekaby, Ahemd K Chellab, Ibraheem Mohammed Ali

**Affiliations:** 1 Neurology Department, Al-Diwaniyah Teaching Hospital, Diwaniyah, IRQ; 2 Neurology Department, University of Mosul, Mosul, IRQ; 3 Neurology Department, Azadi Teaching Hospital, Duhok, IRQ; 4 Stroke Medical Division, Great Western Hospital, Swindon, GBR; 5 College of Medicine, University of Al-Qadisiyah, Diwaniyah, IRQ

**Keywords:** iraq, low- and middle-income country (lmic), stroke epidemiology, stroke management, stroke systems of care

## Abstract

Background

Stroke remains an important cause of death and long-term disability. Contemporary data on acute stroke care in Iraq remain limited.

Objective

This study aimed to describe patient characteristics, vascular risk factors, stroke subtypes, and reperfusion metrics among patients presenting with acute stroke to a thrombolysis-capable tertiary center in Iraq.

Methods

This retrospective observational study analyzed prospectively maintained registry data from consecutive adult patients presenting with acute stroke to the emergency department of Al-Diwaniyah Teaching Hospital in Diwaniyah, Iraq, between October 2023 and September 2024. Demographic characteristics, stroke subtype, vascular risk factors, treatment patterns, and workflow metrics were evaluated.

Results

A total of 339 patients were included. The mean age was 64.1 ± 12.9 years, and 58.1% were male. Acute ischemic stroke accounted for 72% of cases, while intracerebral hemorrhage accounted for 28%. Hypertension was the most common vascular risk factor (70.5%), followed by diabetes mellitus (38.3%) and prior stroke/transient ischemic attack (22.7%). Multiple vascular risk factors were present in 57.5% of patients. Among patients with acute ischemic stroke, 50.8% arrived within 4.5 hours of symptom onset. Twenty-one patients received intravenous thrombolysis, representing 8.6% of all ischemic stroke cases and 16.9% of those arriving within 4.5 hours. The median door-to-needle time was 110 minutes (interquartile range (IQR): 90-140). Two patients underwent mechanical thrombectomy following transfer to another facility.

Conclusions

Patients presenting with acute stroke in this registry exhibited a high burden of modifiable vascular risk factors, and a substantial proportion presented at a relatively young age. Although half of ischemic stroke patients arrived within the therapeutic time window, reperfusion therapy utilization remained limited, and treatment delays were common. These findings identify important opportunities to strengthen stroke systems of care, improve workflow efficiency, and expand access to evidence-based acute stroke therapies in Iraq.

## Introduction

Stroke remains a leading contributor to global morbidity and mortality, ranking as the second most common cause of death and disability worldwide [1,2]. The Global Burden of Disease (GBD) study reported a prevalence of approximately 1,322 per 100,000 population and an annual incidence of 156 per 100,000, with a continued increase in stroke-related mortality and disability-adjusted life years (DALYs) [3,4].

The American Heart Association/American Stroke Association (AHA/ASA) defines stroke as brain, spinal cord, or retinal cell death attributable to ischemia, based on pathological, imaging, or clinical evidence of permanent injury [5]. This definition includes both ischemic and hemorrhagic stroke subtypes and emphasizes neuroimaging confirmation. In contrast, the World Health Organization (WHO) definition is primarily clinical, describing stroke as a rapidly developing focal or global neurological deficit lasting more than 24 hours or leading to death, with no apparent nonvascular cause [6]. Despite advances in neuroimaging, the WHO definition remains widely used in epidemiological studies, particularly in resource-limited settings.

In Iraq, stroke ranks as the third leading cause of death according to the Ministry of Health [7].

Globally, ischemic stroke accounts for approximately 65% of all cases, while intracerebral and subarachnoid hemorrhage (SAH) comprise the remainder [2]. In low- and middle-income countries (LMICs), hemorrhagic strokes are proportionally more common, closely linked to high and often untreated hypertension and weaker health systems [8,9].

A systematic review of 67 studies found the mean age of stroke in LMICs was approximately 5.5 years younger than in high‑income countries (63.1 vs 68.6 years) [10]. Hypertension, diabetes, smoking, and dyslipidemia are the predominant modifiable risk factors driving this epidemic [11].

Reliable stroke registries are essential for effective planning. The WHO STEPS Stroke framework addresses this need, but local data in Iraq remain scarce, and access to acute reperfusion therapy, such as intravenous thrombolysis and mechanical thrombectomy, is limited to select tertiary centers [12,13].

During the study period, access to reperfusion therapies in Iraq remained limited to a small number of specialized centers. Intravenous thrombolysis services were available in selected tertiary hospitals in Sulaymaniyah, Erbil, Baghdad, and Al-Qadisiyah (at the capital city Diwaniyah). Mechanical thrombectomy services were even more limited and were largely concentrated in a few centers, with restricted geographical coverage and availability. These limitations may have contributed to disparities in access to advanced stroke care across the country.

This study aimed to describe patient characteristics, stroke subtypes, vascular risk factors, patterns of acute stroke care, and reperfusion metrics among patients presenting with acute stroke to the only thrombolysis-capable center in Al-Qadisiyah Governorate, Iraq.

## Materials and methods

This retrospective observational study analyzed prospectively maintained stroke registry data from Al-Diwaniyah Teaching Hospital in Diwaniyah, Iraq, between October 1, 2023, and September 30, 2024. Consecutive adult patients (≥18 years) presenting with acute stroke during the study period were screened. Patients with acute stroke confirmed by neuroimaging were included in the analysis.

All patients underwent non-contrast brain CT at presentation. Brain MRI was performed selectively when clinically indicated and available. CT angiography, CT perfusion, MR angiography, transcranial Doppler, and carotid ultrasound were not routinely performed for all patients during the study period and therefore were not systematically analyzed in this registry.

Stroke diagnosis was established based on clinical evaluation and neuroimaging findings. All patients underwent non-contrast brain CT, while brain MRI was performed in selected patients when clinically indicated and available. Patients were categorized as having ischemic stroke or hemorrhagic stroke. Ischemic stroke syndromes were further classified according to the Oxfordshire Community Stroke Project (OCSP) criteria into total anterior circulation infarction (TACI), partial anterior circulation infarction (PACI), lacunar infarction (LACI), and posterior circulation infarction (POCI). OCSP classification was used because it could be consistently applied using routinely available clinical and CT-based data. The Trial of Org 10172 in Acute Stroke Treatment (TOAST) classification was not applied because vascular imaging, echocardiography, prolonged cardiac monitoring, and complete etiological investigations were not systematically available for all patients in this retrospective registry.

Patients were excluded if neuroimaging did not confirm stroke, if the diagnosis was transient ischemic attack (TIA), SAH, cerebral venous thrombosis, traumatic intracranial hemorrhage, subdural hematoma, or another stroke mimic, or if key registry variables were missing.

Data collected included demographic characteristics, vascular risk factors, stroke subtype, and treatment information, and particular emphasis was placed on time metrics related to acute stroke care, including symptom onset-to-arrival time, intravenous thrombolysis utilization, and door-to-needle (DTN) time.

Intravenous thrombolysis was performed using alteplase at a dose of 0.9 mg/kg (maximum dose 90 mg) according to contemporary stroke treatment guidelines. For unwitnessed strokes, symptom onset time was defined using the last-known-well time. Registry variables included symptom onset time, emergency department arrival time, CT timing, and thrombolysis administration time when applicable.

Hypertension was defined as a documented history of hypertension or current use of antihypertensive medication. Diabetes mellitus was defined as a documented diagnosis or current use of glucose-lowering therapy. Dyslipidemia was defined as a documented diagnosis or current use of lipid-lowering medication. Atrial fibrillation was identified from a documented medical history, electrocardiography, or cardiac monitoring records. Smoking status was recorded as current or former tobacco use.

Time metrics included symptom onset-to-arrival time and DTN time for patients treated with intravenous thrombolysis. Reperfusion therapies, including intravenous thrombolysis and mechanical thrombectomy, were recorded.

The study was approved by the Research Committee at the Human Development and Training Center of Al-Diwaniyah Health Directorate, Ministry of Health (approval number: 80). Patient confidentiality was maintained throughout the study.

Statistical analysis

Data were analyzed using IBM SPSS Statistics for Windows, V. 29.0 (IBM Corp., Armonk, NY, USA). Continuous variables were summarized as means and standard deviations (SDs) when normally distributed or medians with interquartile ranges (IQRs) when appropriate. Categorical variables were summarized using frequencies and percentages.

Given the descriptive epidemiological nature of the study, analyses were primarily descriptive and focused on characterizing patient demographics, vascular risk factors, stroke subtypes, treatment patterns, and key workflow metrics. No formal hypothesis testing or multivariable analyses were performed.

## Results

A total of 339 patients with acute stroke were included (58.1% male; mean age 64.1 ± 12.9 years). Nearly half (48.6%) were younger than 65 years. Hypertension was the most common risk factor (70.5%), followed by diabetes mellitus (38.3%) (Table 1).

**Table 1:** Demographic and clinical characteristics of the study cohort (N = 339) TIA: transient ischemic attack

Characteristic	Frequency (%)
Age group	
<65 years	165 (48.6%)
65-79 years	129 (38.1%)
≥80 years	45 (13.3%)
Mean age (±SD)	64.1 (±12.9)
Sex	
Male	197 (58.1%)
Female	142 (41.9%)
M:F ratio	1.38:1
Risk factors	
Hypertension	239 (70.5%)
Diabetes mellitus	130 (38.3%)
Prior stroke/TIA	77 (22.7%)
Obesity	40 (11.8%)
Dyslipidemia	35 (10.3%)
Smoking	57 (16.8%)
Ischemic heart disease	34 (10%)
Atrial fibrillation	17 (5%)
Other risk factors	18 (5.3%)
Risk factor distribution	
Single risk factor	106 (31.3%)
Multiple risk factors	195 (57.5%)
No risk factors	38 (11.2%)

Acute ischemic stroke (AIS) accounted for 72% of cases, with LACI being the most frequent subtype (44.3%) (Table 2). Key acute stroke care metrics demonstrated substantial delays across the care pathway. Although 57.2% of patients resided within 20 km of the hospital, only 50.8% of AIS patients arrived within 4.5 hours of symptom onset. Among AIS patients, 21 received intravenous thrombolysis, representing 8.6% of all AIS cases, and 16.9% of cases arrived within 4.5 hours of symptom onset. Two patients underwent mechanical thrombectomy after transfer to a neurointerventional center in another governorate.

**Table 2:** Stroke types and subtypes (N = 339)

	Frequency (%)
Stroke type	
Acute ischemic stroke (AIS)	244 (72%)
Intracerebral hemorrhage (ICH)	95 (28%)
AIS subtype (N = 244)	
Lacunar infarction (LACI)	108 (44.3%)
Partial anterior circulation infarction (PACI)	77 (31.6%)
Posterior circulation infarction (POCI)	31 (12.7%)
Total anterior circulation infarction (TACI)	28 (11.4%)

The median DTN time was 110 minutes (IQR: 90-140). Two patients underwent mechanical thrombectomy following transfer to another facility (Table 3).

**Table 3:** Strategic access to care and IVT utilization (AIS patients only) AIS: acute ischemic stroke; IVT: intravenous thrombolysis; DTN: door-to-needle; IQR: interquartile range

Parameter	Frequency (%)
Distance from hospital (N = 339)	
≤20 km	194 (57.2%)
>20 km	145 (42.8%)
Arrival time to hospital (N = 244)	
<4.5 hours	124 (50.8%)
≥4.5 hours	120 (49.2%)
AIS patients arriving within 4.5 hours (N = 124)	
Received IVT	21 (16.9%)
Did not receive IVT	103 (83.1%)
Median DTN time	110 minutes (IQR: 90-140)

## Discussion

This study highlights key gaps in acute stroke care in a resource-limited Iraqi setting.

Nearly half of our cohort was under 65 years, highlighting the socioeconomic and public health implications of premature stroke in a working-age population, consistent with LMIC trends [1,10,14].

Hypertension and diabetes were the dominant risk factors, consistent with global figures of modifiable risk factors, emphasizing the urgent need for population-level prevention and chronic disease management strategies [11].

Despite the majority of patients residing within 20 km of the hospital, only 50.8% of ischemic stroke patients arrived within the thrombolysis window. However, this proportion compares favorably with several reports from LMICs, where delayed presentation remains a major barrier to reperfusion therapy. For example, recent studies from India and sub-Saharan Africa reported substantially lower rates of timely hospital arrival among acute stroke patients [15,16]. This relatively favorable rate in our cohort may be partly explained by the small geographic size of the city and the centralized referral pattern, where most patients are directly transported to the single tertiary hospital providing stroke care. These findings suggest that, in our setting, the principal challenge was not solely prehospital delay but also the conversion of early arrival into effective reperfusion therapy.

In our cohort, ischemic stroke accounted for 72% of all stroke cases, consistent with regional observations from the Middle East and North Africa (MENA) and neighboring Asian populations. Although the Safe Implementation of Treatments in Stroke (SITS)-MENA registry reported a comparatively lower proportion of hemorrhagic stroke, this likely reflects the registry's focus on reperfusion-oriented stroke pathways and thrombolysis-eligible patients, resulting in an inherent predominance of ischemic stroke cases. In contrast, our cohort included all stroke subtypes admitted to the center and may provide insight into the distribution of stroke subtypes among patients presenting to a tertiary referral center in Al-Qadisiyah [9,17,18].

In our cohort, not all patients with AIS arriving within 4.5 hours were eligible for intravenous thrombolysis. Some had clinical or imaging-based contraindications, while others became ineligible because of in-hospital delays during evaluation and treatment processes. Patient or family refusal of treatment also contributed to the gap between early arrival and thrombolysis utilization. As this study reflects the early phase of a newly established thrombolysis service, evolving institutional experience and variability in provider familiarity with reperfusion therapy may also have influenced treatment patterns.

Although modest, the intravenous thrombolysis utilization rate (8.6%) is within the range reported by several studies from the Asian settings, where delayed presentation and evolving stroke systems continue to limit reperfusion eligibility [19].

Delays were notable, with median DTN time exceeding current guideline recommendations [20].

The prolonged median DTN likely reflects the early developmental phase of thrombolysis services at our institution during the study period. Several workflow-related factors may have contributed, including delays in patient assessment, neuroimaging acquisition and interpretation, laboratory processing, multidisciplinary coordination, and obtaining informed consent. These findings highlight opportunities for quality improvement initiatives such as streamlined stroke pathways, parallel processing of investigations, dedicated stroke team activation, and continuous staff training.

These findings should be interpreted within the context of a newly established stroke service, as intravenous thrombolysis had not been performed in our center prior to this period. Therefore, the observed delays may reflect the challenges typically encountered during the early developmental phase of organized stroke care. Similar patterns were reported in other countries before the maturation of stroke pathways and implementation of structured quality improvement initiatives [21,22].

Endovascular therapy remains largely inaccessible, with only two patients receiving thrombectomy after significant delays. Expanding regional neurointerventional capacity is essential.

Study limitations

Our study has several limitations. As a single-center hospital-based registry, the findings should not be interpreted as population-level estimates for the governorate or Iraq as a whole.

In addition, stroke severity was not systematically assessed using standardized measures such as the National Institutes of Health Stroke Scale (NIHSS), limiting the evaluation of baseline neurological deficits. Similarly, functional outcomes, including the modified Rankin scale (mRS), were not routinely collected, precluding the assessment of post-stroke disability and recovery. Post-thrombolysis mortality and symptomatic intracranial hemorrhage were also not systematically recorded in the registry. These limitations restrict the evaluation of treatment effectiveness and clinical outcomes beyond the acute hospitalization period.

Additional limitations should be acknowledged. The TOAST classification could not be performed because comprehensive etiologic investigations were not systematically available for all patients. Advanced vascular imaging modalities were not routinely available during the study period. Furthermore, door-in-door-out time was not systematically recorded for patients transferred for mechanical thrombectomy.

## Conclusions

This hospital-based stroke registry provides one of the first contemporary descriptions of acute stroke patients presenting to a thrombolysis-capable center in Al-Qadisiyah, Iraq. Patients presented at a relatively young age and exhibited a high burden of modifiable vascular risk factors, particularly hypertension and diabetes mellitus, highlighting the ongoing need for population-level prevention and improved cardiovascular risk factor control.

Although approximately half of ischemic stroke patients arrived within the therapeutic time window, utilization of intravenous thrombolysis remained limited, and DTN times exceeded recommended targets, indicating important gaps in both prehospital and in-hospital stroke pathways. These findings suggest that improvements in public awareness, emergency medical services, stroke recognition, pre-notification systems, and workflow optimization may have substantial potential to increase access to reperfusion therapy.

The substantial proportion of intracerebral hemorrhage observed in this cohort further emphasizes the need for better hypertension detection and control strategies. In addition, the very limited access to mechanical thrombectomy highlights ongoing disparities in advanced stroke care within resource-constrained settings.

Overall, these data establish an important local benchmark for stroke care in Al-Qadisiyah and may serve as a foundation for future quality improvement initiatives, multicenter stroke registries, and population-based studies aimed at strengthening stroke systems of care in Iraq.
